# CMC-Based Injectable Hydrogels Crosslinked by Diels–Alder Chemistry for Wound Healing Applications

**DOI:** 10.3390/gels11090674

**Published:** 2025-08-23

**Authors:** Israr Ali, Urwa Shahid, Seon-Hwa Kim, Suganthy Ramamoorthy, Won Han, Minseon Kim, Vishal Gavande, Won-Ki Lee, Joong Ho Shin, Sang-Hyug Park, Kwon Taek Lim

**Affiliations:** 1 Department of Smart Green Technology Engineering, Pukyong National University, Busan 48513, Republic of Korea; israrchem@gmail.com; 2 Industry 4.0 Convergence Bionics Engineering, Pukyong National University, Busan 48513, Republic of Korea; urwashahid6@gmail.com (U.S.); seonhwakim431@gmail.com (S.-H.K.); ramsuga30@gmail.com (S.R.); hanwon0427@naver.com (W.H.); minseonida1@naver.com (M.K.); jhshin@pknu.ac.kr (J.H.S.); 3 Department of Materials Science and Engineering, Saarland University, 66123 Saarbrucken, Germany; vgawande77@gmail.com; 4 Department of Polymer Engineering, Pukyong National University, Busan 48513, Republic of Korea; wonki@pknu.ac.kr; 5 Major of Biomedical Engineering, Division of Smart Healthcare, College of Information Technology and Convergence, Pukyong National University, Busan 48513, Republic of Korea; 6 Institute of Display Semiconductor Technology, Pukyong National University, Busan 48513, Republic of Korea

**Keywords:** carboxymethyl cellulose, injectable hydrogel, curcumin, drug release, wound healing

## Abstract

Chronic wounds disrupt natural healing and tissue regeneration, posing a major challenge in healthcare. Conventional wound care often lacks effective drug delivery, tissue integration, infection control, and patient comfort. However, injectable hydrogels offer localized, minimally invasive treatment and conform to irregular wound shapes. This study presents carboxymethyl cellulose (CMC)-based injectable hydrogels, prepared via Diels–Alder click chemistry using highly furan functionalized CMC (45%) and a bismaleimide crosslinker. The hydrogels showed a rapid gelation time (<490 s) under physiological conditions. The hydrogel exhibited favorable physicochemical and mechanical properties, as well as sustained curcumin release (∼80% in 5 days). In vitro studies confirmed excellent biocompatibility with NIH3T3 fibroblasts and notable antibacterial activity against *E. coli*, supporting its potential for wound healing applications.

## 1. Introduction

Chronic wounds affect about 3% of U.S. adults over 65, and the global rise in diabetes (projected to reach 700 million by 2045) further elevates wound-related risks [1,2]. Chronic wounds arise when the normal healing process is disrupted or prolonged, often due to underlying physiological factors such as diabetes, persistent oxidative stress from reactive oxygen species (ROS), or ongoing bacterial infections that prolong inflammation [3]. While many common wounds can generally heal and return to their original state over time, the wound healing process does not always proceed in a flawless, orderly manner [4]. Wound repair involves four interconnected stages: hemostasis, inflammation, proliferation, and remodeling. Disruptions in cellular and tissue repair can hinder proper healing [5], causing persistent infections [6], abnormal wound closure, excessive inflammation [7], and other healing challenges [8].

Maintaining controlled conditions at the wound site is crucial to support the complex cellular activities necessary for effective healing. Wound dressings form a protective layer over the wound, preventing external infections and significantly aiding in the healing process [9]. A suitable wound dressing should balance effectiveness, patient safety, and cost-efficiency. To ensure a conducive healing environment, the ideal wound dressing should be both biodegradable and biocompatible, with optimal permeability for water vapor [10,11]. Additionally, it must adapt the wound shape, offer protection from microbial invasion, and withstand mechanical and thermal stresses, all while managing moisture levels effectively [4,12]. Among the most widely used materials for wound dressings are hydrogels [13,14], films [15], foams [16,17], and hydrocolloids [18,19].

Hydrogels, with their highly crosslinked hydrophilic polymeric network structure, have recently drawn significant interest for wound healing due to their remarkable water wicking property and good biocompatibility, mimicking the natural extracellular matrix [2] with excellent hydrophilic properties [14]. In addition to providing a framework for cell growth, hydrogels can absorb exudate and maintain a moist wound environment, which aids in the healing process. Various natural polymers―such as chitosan [20], alginate [21], hyaluronic acid [22], collagen [23], and gelatin [24]―are commonly used to develop hydrogels for wound healing applications. Polymer-based drug delivery systems commonly face challenges such as fast degradation and the poor bioavailability of natural compounds, limiting their overall therapeutic effectiveness. Among various polymers, carboxymethyl cellulose (CMC), a derivative of cellulose, emerges as a highly suitable candidate for drug delivery matrices. This is attributed to its favorable characteristics, including biocompatibility, non-toxicity, water solubility, biodegradability, moisture retention, adjustable rheological properties, stability, and minimal immunogenic response. The backbone of CMC, which contains carboxylate and hydroxyl groups, has been chemically modified with various functional side groups, such as methacrylate, alkyne/azide, aldehyde, and thiol, improving applicability for cancer treatment [25,26]. CMC has been utilized in wound healing applications due to its biocompatibility, biodegradability, structural similarity to natural tissue, cost-effectiveness, and non-toxicity [27,28].

The conventional hydrogels were pre-formed and required invasive surgical methods for application to the target sites. However, recent advances have led to the development of injectable hydrogels, where hydrogel precursors are delivered through a syringe and crosslinked in situ [29] using various chemical reactions, such as photo crosslinking [30], Diels–Alder (DA) reactions [31], and inverse electron demand DA reactions. Compared to conventional hydrogels, in situ formed injectable hydrogels prepared through chemical crosslinking offer several additional advantages, including lower implantation costs, reduced patient discomfort with minimal invasiveness, and improved tissue regeneration at the target site. This property makes them suitable for use in various shapes and sizes, allowing for easy, off-the-shelf treatment of wounds [32,33].

Furthermore, injectable hydrogels can administer drugs at the localized wound site, which helps accelerate healing.

Curcumin, a natural polyphenol derived from plants, is widely used in the treatment of inflammatory and chronic diseases. The combination of antimicrobial, antifungal, anti-inflammatory, and antioxidant effects highlights the strong potential of this compound in supporting wound healing [34]. However, its therapeutic efficacy is limited by poor water solubility. Injectable hydrogels can help overcome this limitation by encapsulating curcumin and allowing controlled release directly at the wound site [30].

**Scheme 1:**
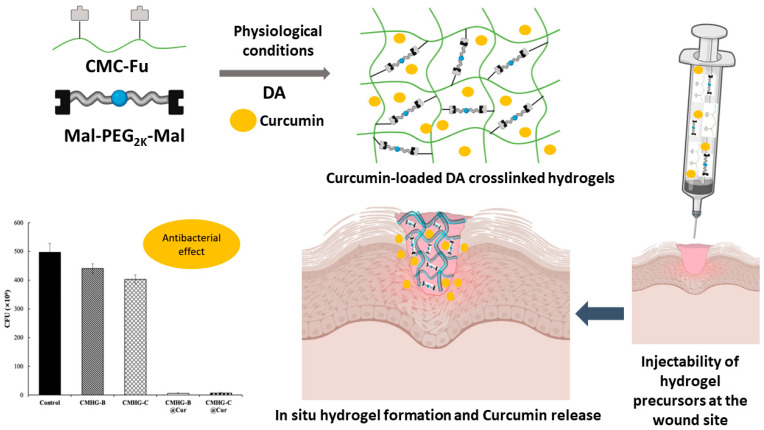
Graphical illustration of preparation of CMC-based injectable hydrogels loaded with curcumin via DA click reaction for wound dressing applications.

This study aims to synthesize injectable hydrogels loaded with curcumin for controlled release at the wound site, promoting an effective healing process as depicted in Scheme 1. For this purpose, CMC—a biodegradable, water-soluble biopolymer obtained from cellulose—was selected owing to its wide-ranging biomedical uses, such as wound repair, controlled drug delivery, and tissue engineering [26]. First, it was functionalized with furan (Fu) using 4-(4,6-dimethoxy-1,3,5-triazin-2-yl)-4-methyl morpholinium chloride (DMTMM) chemistry, followed by curcumin addition to achieve a homogeneous solution. Finally, a PEG maleimide crosslinker (Mal-PEG_2K_-Mal) was applied, enabling the rapid formation of curcumin-loaded injectable hydrogels via DA chemistry under physiological conditions. Swelling ratios, morphology, and rheological properties were analyzed to determine the physical and mechanical characteristics of the synthesized hydrogels. Over 80% of curcumin was released from the hydrogel within 5 days in physiological environments. In vitro tests demonstrated its non-toxicity toward NIH 3T3 fibroblast cells and improved antibacterial activity against *E. coli* bacteria due to the presence of curcumin. The hydrogels demonstrated significant potential for offering minimally invasive delivery, controlled drug release, and better patient outcomes, marking a promising step forward in wound care therapies.

## 2. Results and Discussion

### 2.1. Synthesis of Fu Conjugated Carboxymethyl Cellulose (CMC-Fu)

**Figure 1:**
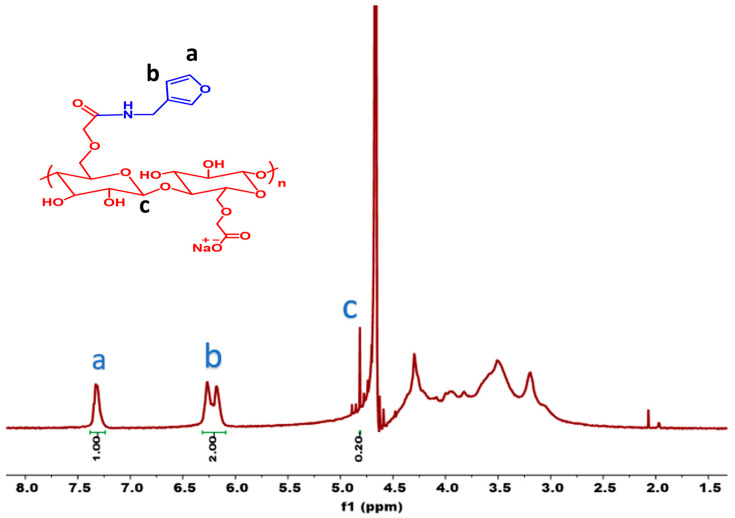
^1^H NMR spectrum of CMC-Fu.

**Figure 2:**
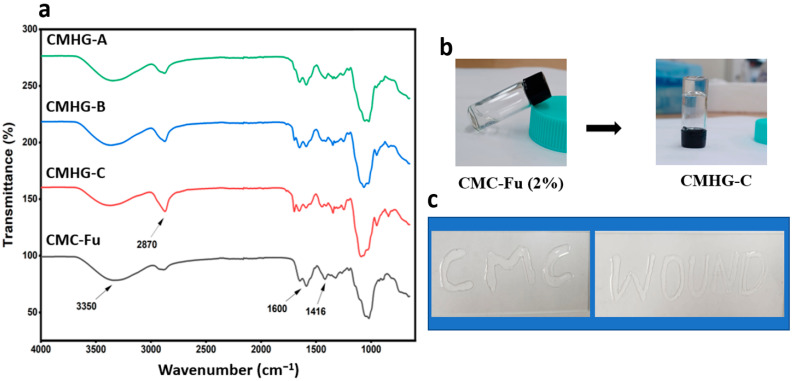
(**a**) FTIR spectrum of CMC-Fu, CMHG-C, CMHG-B, and CMHG-A. (**b**) Visual documentation of hydrogel gelation. (**c**) Visual representation of injectability of CMC hydrogels.

In this work, CMC was chemically conjugated with Fu by reacting the COOH groups with the NH_2_ groups of Fu-NH_2_ (furfuryl amine, FA) using DMTMM-mediated coupling, resulting in a precursor suitable for the DA reaction. The modification of CMC-Fu was confirmed through analysis using the ^1^H NMR spectrum (Figure 1). The proton signals observed in the range of 6.18 to 7.48 ppm were associated with the FA groups, whereas the proton peak around 4.9 ppm was likely due to the proton attached to the β-(1→4) glycosidic bonds in the backbone of CMC [26,35]. The degree of substitution (DS) of Fu was calculated by comparing the integrals of the Fu with glycosidic bonds, and it was found to be 45%. Further, FTIR analysis was conducted to validate the structure of CMC-Fu (Figure 2a). A broad absorption band appeared at 3400 cm^−1^ was attributed to the OH groups present in CMC. Additionally, absorption peaks near 1600 cm^−1^ and 1409 cm^−1^ were associated with stretching vibrations of C=O and bending vibrations of N–H, respectively. The presence of characteristic peaks at 1145 is attributed to the stretching vibrations of C–N of Fu, whereas the small peaks of the Fu CH bonds were noted at 916 + 728 cm^−1^, confirming the successful synthesis of CMC-Fu.

### 2.2. Fabrication of Hydrogels and Rheological Analysis

The use of Fu-Mal DA chemistry offers notable advantages, including mild, catalyst-free conditions and spontaneous reactivity in water. The aqueous environment accelerates the reaction, making it ideal for precisely tuning hydrogel structures to achieve fast-responding, functional hydrogels [36]. To enable the Fu-Mal reaction, Mal-PEG_2K_-Mal was used as a crosslinker with CMC-Fu, facilitating the facile synthesis of injectable hydrogels. Three types of hydrogels were prepared using different crosslinker ratios: 10:10, 10:5, and 10:2.5 (CMHG-C, CMHG-B, and CMHG-A).

**Figure 3:**
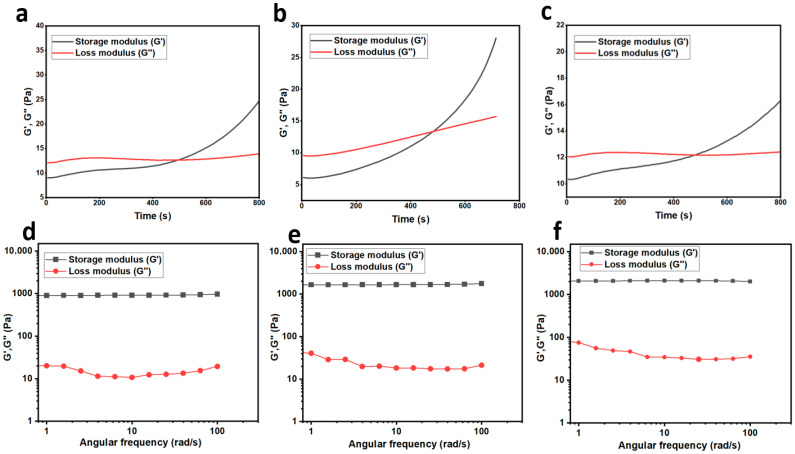
Storage and loss moduli of CMHG- (A, B, and C) hydrogels, (**a**–**c**) plotted against step time, and (**d**–**f**) plotted against angular frequency.

Visual determination of the sol-gel transition of hydrogels was carried out by the vial inversion method, as evident from the photographs (Figure 2b). According to the tube inversion test, the 10:10 hydrogel demonstrated a quick gelation time of less than 10 min, attributed to the rapid Fu/Mal reaction. Rheology is an essential tool for evaluating the viscoelastic properties of hydrogels. Especially, it measures the gelation kinetics (sol-gel transition) and injectable character of hydrogels by measuring the elastic modulus (G′) and shear modulus (G″). CMC hydrogel gelation time and viscoelasticity were measured by the rheometer-based time and frequency-dependent tests on storage (G′) and loss (G″) moduli. Figure 2c presents visual evidence of the injectability of the CMC hydrogels. As shown in Figure 3a–c, all three hydrogel formulations completed gelation within 500 s, further demonstrating their potential for injectable applications.

Mechanical properties are vital for hydrogel-based drug delivery, as they influence drug loading, release rates, and biocompatibility by ensuring structural integrity and enabling controlled release in vivo. As shown by Figure 3d–f, under increasing angular frequencies from 0 to 100 rad/s in the frequency sweep test, hydrogels CMHG-A, CMHG-B, and CMHG-C demonstrated the storage modulus of 1000 Pa, 1700 Pa, and 2100 Pa respectively. The observed storage modulus values indicate that the hydrogels exhibit mechanical properties within a range compatible with human skin, ensuring structural suitability for wound healing applications [37]. Additionally, an increase in crosslinker concentration enhances the mechanical strength of the hydrogels due to the formation of denser and more compactly crosslinked polymer networks [38].

**Figure 4:**
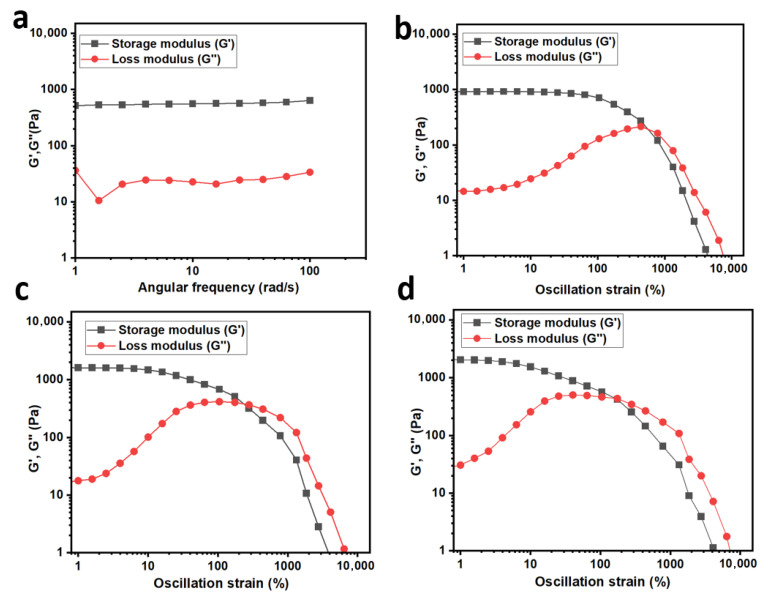
(**a**) Storage and loss modulus of CMHG-B hydrogel as a function of angular frequency after immersing the sample in PBS over 3 days. (**b**–**d**) Dynamic oscillatory strain amplitude sweep of CMHG-A, CMHG-B, and CMHG-C hydrogels at 10 rad/s.

The mechanical stability of the CMHG-B hydrogel under physiological conditions was assessed by incubating the samples in PBS at 37 °C for 3 days, followed by rheological analysis. As shown in Figure 4a, both the storage modulus (G′) and loss modulus (G′′) decreased significantly over this period, indicating a gradual weakening of the hydrogel network. This reduction is likely due to the partial hydrolytic degradation and swelling of the hydrogel matrix under aqueous conditions. Nevertheless, the hydrogel retained sufficient structural integrity to provide effective wound protection and enable controlled drug release during the initial period of application.

Dynamic oscillatory strain amplitude sweep measurements were performed over a strain range of 0.1% to 10,000% at a constant frequency of 10 rad/s. As shown in Figure 4b–d, the rheological profiles of all three hydrogel samples demonstrate typical viscoelastic behavior. At low strains, the storage modulus (G′) predominates, indicating solid-like characteristics, while a crossover with the loss modulus (G″) occurs at higher strains, reflecting network disruption and a transition toward liquid-like behavior under large deformation. The progressive increase in crossover strain from CMHG-A to CMHG-C suggests enhanced mechanical stability and strain tolerance, likely due to higher crosslinking density in the latter samples.

### 2.3. Swelling Studies of Hydrogels

**Figure 5:**
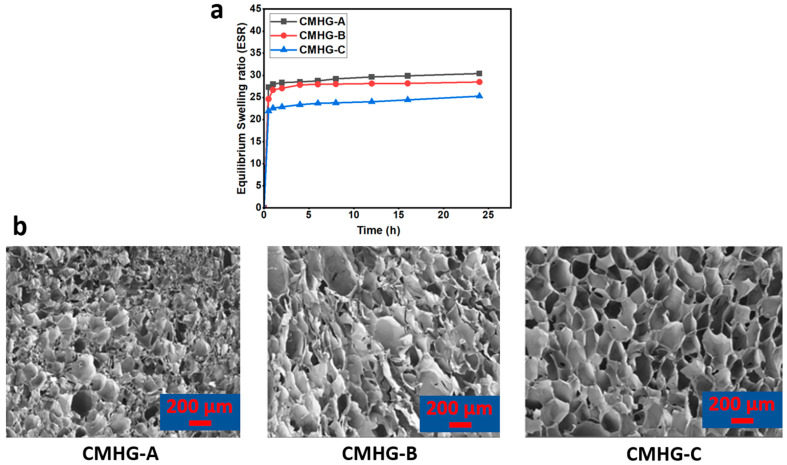
(**a**) Swelling ratio of hydrogels in physiological conditions. (**b**) Morphology of CMHG- (A, B, and C) hydrogels.

The swelling behavior of hydrogels is essential in drug delivery, as it controls release kinetics, improves drug diffusion, and supports the development of stimuli-responsive systems that respond to environmental triggers such as pH or temperature for targeted therapy. As shown in Figure 5a, the swelling ratio of the hydrogels decreased as the crosslinker concentration increased, with CMHG-A exhibiting the highest swelling ratio and CMHG-C the lowest. The reduced swelling ratio with increased crosslinker concentration is typical for hydrogels, likely due to tighter networks with smaller mesh sizes and restricted macromolecular chain mobility, limiting water uptake [39].

### 2.4. Morphologies of Hydrogels

FE-SEM analysis was performed to investigate the morphology of CMC hydrogels after lyophilization, revealing their porous structure formed by the removal of internal water droplets. As shown in Figure 5b, the hydrogel samples exhibited a well-defined three-dimensional porous structure with interconnected pores. Notably, the CMHG-C hydrogel, with a mean pore size of 160 ± 34 µm, displayed a more uniform and well-defined porous network compared to the CMHG-A hydrogel, which had a mean pore size of 100 ± 25 µm, likely due to its higher crosslinking density. The hydrogel’s network structure facilitates substantial water uptake, thereby enhancing its swelling capacity and drug-loading efficiency [36]. Additionally, a dense network plays a critical role in wound healing by improving mechanical stability, offering a barrier against infection, maintaining a moist environment, and supporting key cellular functions involved in tissue regeneration.

### 2.5. Drug Loading and Release Studies

**Figure 6:**
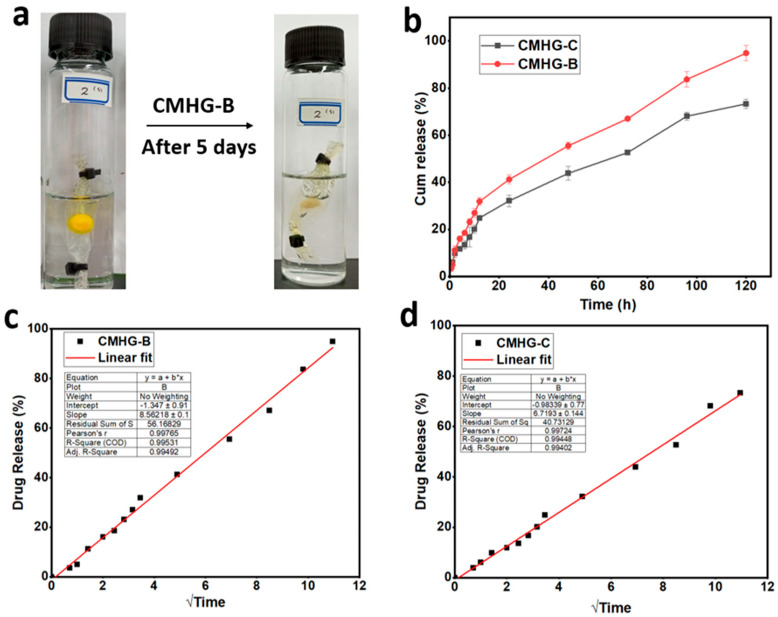
(**a**) Visual representation of drug release from CMHG-B hydrogels. (**b**) Cumulative curcumin release graphs of CMHG-B and CMHG-C hydrogels in PBS (pH 7.4). (**c**,**d**) Percentage release of drug plotted as a function of the square root of time according to Higuchi model.

Curcumin was used in this study as a model drug to investigate its loading efficiency and release profile in the two hydrogels CMHG-B and CMHG-C in PBS solution at pH 7.4. The loading efficiency of curcumin in CMHG-B was 89.5%, slightly lower than that of CMHG-C, which reached 92%. These findings align with expectations, suggesting that denser network structures enhance drug loading capacity. This indicates that the density of the hydrogel network plays a key role in determining drug loading efficiency [35]. Figure 6a visually illustrates the drug release, showing that curcumin was gradually released from the hydrogel over 5 days, as indicated by the fading of the yellowish color.

The graphs in Figure 6b illustrate the curcumin release profiles for five days. By the end of this period, CMHG-C released approximately 75% of the drug, whereas CMHG-B exhibited a higher release, reaching 89%. In this case, drug release from the CMC hydrogels is primarily governed by diffusion. CMHG-B, with its looser network structure and higher swelling capacity, allows for faster drug diffusion, resulting in a higher release rate. In contrast, CMHG-C, which has a denser, more highly crosslinked network and lower swelling, exhibits a comparatively slower release of curcumin.

In addition to the cumulative release profile shown in Figure 6b, the drug release mechanism was further evaluated by plotting the percentage release as a function of the square root of time (t^1/2^), according to the Higuchi model [40]. The resulting linear plot (R^2^ ≈ 0.99) confirms that the release from the CMC hydrogels is predominantly governed by diffusion (Figure 6c,d).

### 2.6. In Vitro Cytocompatibility of Hydrogels

**Figure 7:**
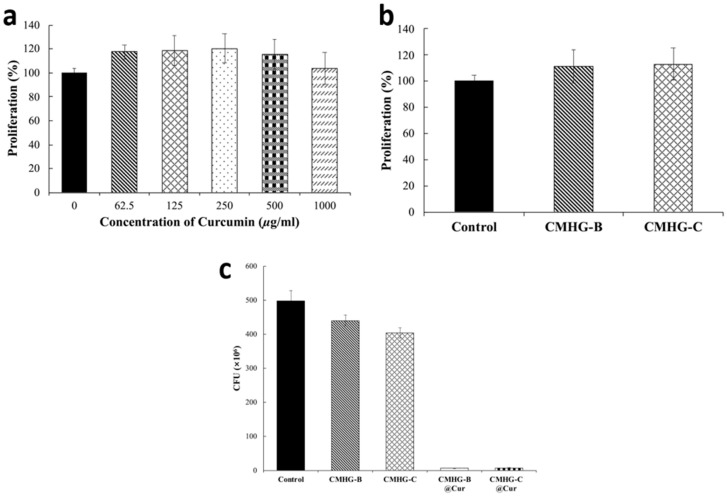
The viability of NIH-3T3 cells after being incubated with (**a**) various concentrations of curcumin and (**b**) CMHG hydrogels. (**c**) Determination of colony-forming unit (CFU) of CMHG and CMHG@Cur hydrogels using *E. Coli* bacteria. *(n* = 4).

Cytotoxicity testing is essential for hydrogels used in wound healing as it ensures that the synthesized material is non-toxic to cells, thereby supporting cell viability, tissue regeneration, and effective wound repair without causing inflammation or delayed healing. In this study, first the cytotoxicity of curcumin was assessed at various concentrations, all of which maintained 100% cell viability, confirming its biocompatibility as a therapeutic component in wound healing (Figure 7a). Similarly, the cytotoxicity of CMHG-B and CMHG-C hydrogels was evaluated, and as shown in Figure 7b, both formulations exhibited cell viability above 100%, likely due to higher porosity of hydrogels fostering improved cell growth and creating a supportive environment within the hydrogel matrix [41], demonstrating their biosafety for wound healing applications. The observed results are consistent with the biocompatible properties of the biopolymer CMC, as documented in the literature [30,42]. Based on these results, the proposed hydrogel system demonstrates strong potential as a promising candidate for biomedical applications, owing to its excellent biocompatibility.

### 2.7. Anti-Bacterial Analysis of Hydrogels

The antibacterial efficacy of both blank hydrogels and curcumin-loaded hydrogels was investigated through in vitro assays employing *Escherichia coli* O157:H7 (ATCC 35150). This particular bacterial strain was selected owing to its relevance as a Gram-negative pathogen commonly associated with wound infections [43], thereby serving as an appropriate model for evaluating the therapeutic potential of the hydrogel formulations. Bacterial colony formation was used to evaluate the antibacterial performance of the hydrogel samples. As shown in Figure 7c, CMHG-C@Cur and CMHG-B@Cur exhibited bacterial colony counts of 8 (×10^6^) and 5 (×10^6^), respectively, after 12 h, underscoring the significant role of curcumin in enhancing the antibacterial efficacy of the hydrogels. In contrast, blank hydrogels without curcumin demonstrated a very high number of bacterial colonies, further emphasizing curcumin’s essential contribution to antimicrobial performance. This enhanced activity may be attributed to the effective release and bioavailability of curcumin, which disrupts bacterial membranes and inhibits protein synthesis [44], ultimately leading to a reduction in bacterial growth. These findings demonstrate that the curcumin-loaded CMHG hydrogel system possesses effective antimicrobial properties capable of preventing bacterial infections in wounds while promoting healing [30,45]. This underscores the hydrogel’s potential as an effective drug delivery system for wound treatment.

### 2.8. In Vitro Biodegradation Analysis of Hydrogels

**Figure 8:**
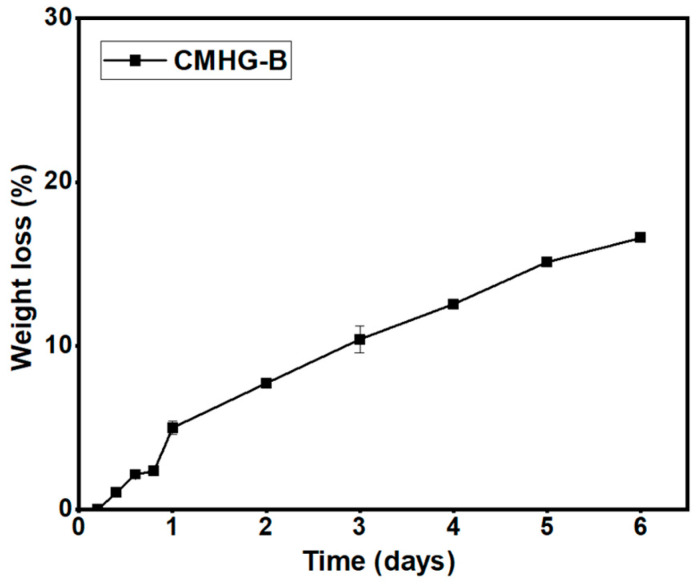
The in vitro biodegradation of CMHG-B hydrogels in PBS (pH 7.4).

Biodegradable hydrogels offer significant advantages for drug delivery, allowing precise control over drug stability and release rates. As shown in Figure 8, the CMHG-B hydrogel exhibited approximately 17% weight loss under physiological conditions over 6 days, generating non-toxic byproducts expected to be biocompatible. These results indicate that the hydrogel maintains sufficient structural integrity to enable controlled therapeutic delivery while safely degrading in a biological environment.

## 3. Conclusions

In this study, injectable CMC-based hydrogels were prepared via DA chemistry using a maleimide crosslinker. NMR and FTIR confirmed Fu functionalization of CMC with high DS (45%). Rapid gelation kinetics (<490 s), validated by time-sweep rheology, demonstrate its suitability for injectable applications. The rheological analysis revealed an appropriate storage modulus suitable for wound healing applications. SEM analysis demonstrated a dense, interconnected network structure, while swelling studies indicated that the hydrogel exhibits a favorable swelling ratio under physiological conditions. Furthermore, the hydrogels demonstrated sustained release of curcumin, with approximately 80% of the drug released over five days under physiological conditions. The hydrogels exhibited no cytotoxicity toward NIH 3T3 cells, while the curcumin-loaded hydrogels demonstrated significantly enhanced antibacterial properties against *E. coli* bacteria. The developed injectable hydrogel system shows great promise for drug delivery in wound healing applications, attributed to its rapid gelation, excellent biocompatibility, and potent antibacterial properties.

## 4. Material and Methods

### 4.1. Materials

Sodium carboxymethyl cellulose (Na-CMC) (n = approx. 1050) (M_w_ ~ 276 kDa), furfuryl amine (FA), and 4-(4,6-dimethoxy-1,3,5-triazin-2-yl)-4-methyl morpholinium chloride (DMTMM) were purchased from Tokyo Chemical Industry (TCI) (Tokyo, Japan). 2-Morpholinoethanesulfonic acid (MES; 99%) buffer (100 mmol/L, pH 5.5) was obtained from Sigma-Aldrich (Seoul, Republic of Korea). 2-Arm-PEG2000-Mal (Mal-PEG_2K_-Mal) was bought from Biopharma PEG Scientific Inc. (Watertown, MA, USA). All other organic chemicals or reagents were obtained from Duksan Pure Chemicals (Ansan, Republic of Korea).

### 4.2. Measurements

^1^H NMR spectra were recorded using a JEOL NMR spectrometer (JNM ECZ-400, JEOL, Akishima-Shi, Japan). FTIR spectra were acquired with an Agilent CARY 640 spectrometer (Agilent Technologies, Santa Clara, CA, USA). UV–Vis measurements were performed using a UV–Vis spectrophotometer (Optizen POP, Optizen, Daejon, Republic of Korea). The hydrogel morphology was observed with a field emission scanning electron microscope (FE-SEM, MIRA 3 system, TESCAN, Brno, Czechia). The viscoelastic properties of the hydrogels were evaluated at 25 ± 0.1 °C using a Discovery HR-2 hybrid rheometer (ARES-G2M, TA Instruments, New Castle, DE, USA) fitted with an 8 mm parallel horizontal plate geometry.

### 4.3. Methods

#### Functionalization of CMC-Fu

CMC-Fu derivative was synthesized by functionalizing CMC with FA groups by using a DMTMM coupling agent. Typically, 1 g (0.0036 mmol) of Na-CMC was dissolved in 100 mL of MES buffer and the contents were purged with N_2_ gas with constant stirring. Afterwards, DMTMM (1.05 g, 3.81 mmol) (pre-dissolved in 5 mL of MES buffer) was injected into the above solution by using a glass syringe. After 1 h of stirring, FA (0.37 g, 3.81 mmol) was introduced into the reaction mixture and the contents were stirred for 24 h at RT. Subsequently, the mixture was precipitated into the excess amount of acetone. The crude CMC-Fu solid was dried under vacuum, rehydrated to a 1% *w*/*v* solution in deionized (DI) water, and dialyzed in abundant of DI water for 4 days. Finally, the refined CMC-Fu was recovered by lyophilization (DS = 45%, experimentally determined by NMR spectrum).

### 4.4. Formation of Hydrogels via DA Reaction

**Scheme 2:**
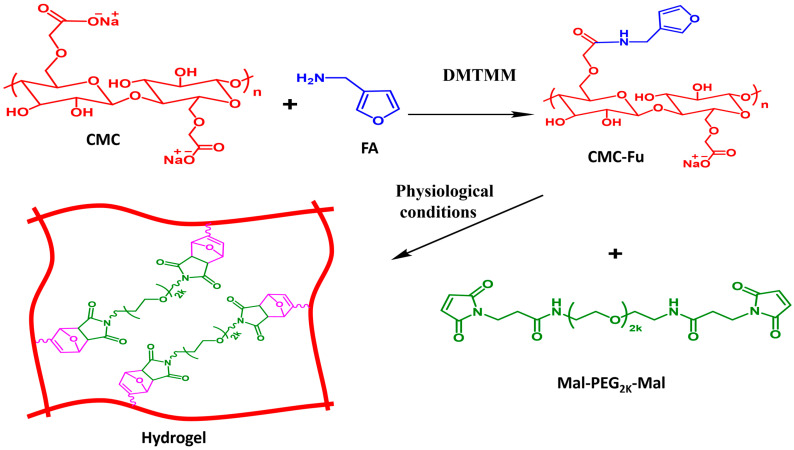
Schematic representation of the preparation of CMC-based hydrogels through DA reaction.

DA crosslinked hydrogels were formulated by a simple one-step mixing of CMC-Fu with the Mal-PEG_2K_-Mal crosslinker as shown in Scheme 2. To assess the physiochemical properties of hydrogels, three different crosslinker compositions were employed to prepare the hydrogels, as shown in Table 1. Briefly, a solution of CMC-Fu (2% *w*/*v*) was gently mixed with various stoichiometric amounts of Mal-PEG_2K_-Mal (10/2.5, 10/5, and 10/10 Fu/Mal) (prepared in PBS) and allowed to form a gel at physiological temperature 37 °C. The tube inversion method was performed to note the gelation time by using a digital stopwatch while inverting the tube, and it was assessed whether the solution flowed or changed into a gel state.

### 4.5. Rheological Analysis of Hydrogels

The viscoelastic behavior of hydrogels was assessed by using a Discovery HR-2 hybrid rheometer (TA instruments) equipped with a parallel plate geometry (20 mm in diameter and 250 µm gap) at 25 ± 0.1 °C. The angular frequency sweep test was performed over a range of angular frequency from 0 to 100 rad/s. In a step-rate time sweep analysis, the measurements were recorded at a continuous strain of 1% and an angular frequency of 10 rad/s. Thereafter, dynamic oscillatory strain amplitude sweep measurements were conducted at ascending strains ranging from 0.1 to 10,000% at a constant frequency of 10 rad/s.

### 4.6. Swelling Study

The equilibrium swelling ratio (ESR) of the hydrogels was determined using a simple gravimetric method. The lyophilized hydrogels were immersed in PBS (pH 7.4) and allowed to swell. After a predetermined time, the hydrogels were taken out, the water was swiped from the surface using a filter paper, and the weight was measured in the swollen state. The procedure was repeated until the weight of the swollen hydrogel became constant. The ESR value was calculated by using the Equation (1).

(1) $$ESR\left(\%\right)=\frac{W_{s}-W_{d}}{W_{d}}\times 100$$

where W_s_ and W_d_ are the weights (mg) of the swollen state and dry state of the hydrogels, respectively.

### 4.7. Curcumin Encapsulation and Release Studies

To evaluate the drug release profile of hydrogels, curcumin-loaded hydrogels (*n* = 3) were tested in phosphate-buffered saline (PBS) at pH 7.4. For sample preparation, a 2% CMC-Fu-curcumin solution was formulated by dissolving 20 mg of CMC-Fu and 1 mg of curcumin in 1 mL of DI water, with continuous stirring to ensure uniformity. Subsequently, 200 μL of the resulting curcumin-polymer mixture was combined with 30 μL of a Mal-PEG_2K_-Mal solution (prepared in different ratios: 10:10 and 10:5) in an Eppendorf tube. The mixture was gently vortexed for 15 s, then incubated at 37 °C for 20 min to allow hydrogel formation. The formed hydrogels were then subjected to freeze-drying. Curcumin-loaded, freeze-dried hydrogels were then washed with a 20% ethanol solution to eliminate unbound drug, and DLE% was assessed by UV-Vis analysis of the supernatant at 430 nm using a standard curve.

Hydrogels loaded with curcumin were placed in dialysis bags (3.5 K MWCO) containing 2 mL PBS and immersed in 30 mL of release medium (20% ethanol, 80% PBS). The samples were placed under shaking conditions at 37 °C. 1 mL of the medium was sampled every few hours, and fresh PBS was replenished. Released curcumin from the hydrogel samples was quantified using UV-Vis spectroscopy at 430 nm.

### 4.8. In Vitro Cytotoxicity

The cytotoxicity assay of curcumin was conducted using NIH 3T3 fibroblasts from KCLB (Korean Cell Line Bank). The cells were cultured under a CO_2_ incubator that maintained 37 °C with high glucose DMEM (Gibco, New York, NY, USA), 10% fetal calf serum (Gibco), and 1% AA (Gibco). Cells were initially seeded in 48-well culture plates at a density of 50,000 cells per well and incubated for 24 h. Subsequently, the cells were treated with fresh medium containing curcumin at concentrations of 0, 62.5, 125, 250, 500, and 1000 µg/mL for 24 h. After treatment, the culture medium was removed, and the WST assay solution was added to assess cell proliferation. The following formula was used to calculate the relative proliferation rate (Equation (2)).

[Relative proliferation(viability) rate (%) = (OD_Control_ − OD_Experiment_)/(OD_Control_) × 100] (2)

The cytotoxicity of CMHG-B and -C (*n* = 4) was performed in cultured NIH 3T3 cells. The previously described procedures were conducted under the specified culture conditions. For the hydrogel assay, cells were seeded in 48-well plates at the same density (50,000 cells per well). Hydrogels were then placed onto transwell inserts and incubated with the cells for 24 h.

### 4.9. Anti-Bacterial Analysis

To examine the anti-bacterial effect of curcumin-loaded CMHG, the broth microdilution method was conducted using *E. coli* O157:H7 (ATCC 35150). The hydrogel samples (n = 4) were incubated at 37 °C with a 50% ethanol solution for 24 h, and then the extracts were collected for testing. The 180 µL of bacterial suspension (10^5^ CFU) and 20 µL of extracts were added to the sterile 96-well plate. The final volume of each well was 200 µL. A culture medium and a bacterial suspension were used to run the control group, while hydrogel extracts were added to the other groups. All the samples were incubated at 37 °C for 12 h to stimulate the growth of bacterial colonies. The wells were counted manually for the determination of the colony-forming unit (CFU).

### 4.10. In Vitro Biodegradation Study

For in vitro biodegradation analysis, CMHG-B hydrogels (*n* = 3) were prepared and incubated in PBS (pH 7.4) at 37 °C for 6 days. The percentage weight loss of the hydrogels was determined using the following equation:

(3) $$\;Weight\;loss\left(\%\right)=\frac{x_{1-}x_{2}}{x_{1}}\times 100$$

where *x*_1_ represents the initial weight of the hydrogels, and *x*_2_ denotes the weight of the degraded hydrogels at each time point.

## Figures and Tables

**Table 1:** Composition and gelation time of hydrogels.

Sample	Fu/Mal Molar Ratio	Gelation Time (s) ^a^
CMHG-A	10/2.5	490
CMHG-B	10/5	477
CMHG-C	10/10	465

^a^ Gelation time was determined from a rheometer by the intersection of G′ and G″.

## Data Availability

The data presented in this paper are available from the corresponding author upon request.
